# MiRNA-disease interaction prediction based on kernel neighborhood similarity and multi-network bidirectional propagation

**DOI:** 10.1186/s12920-019-0622-4

**Published:** 2019-12-23

**Authors:** Yingjun Ma, Tingting He, Leixin Ge, Chenhao Zhang, Xingpeng Jiang

**Affiliations:** 1 School of Mathematics & Statistics, Central China Normal University, Wuhan, 430079 Hubei China; 2 School of Computer, Central China Normal University, Wuhan, 430079 Hubei China; 3 Hubei Provincial Key Laboratory of Artificial Intelligence and Smart Learning, Central China Normal University, Wuhan, 430079 Hubei China; 4 School of Life Sciences, Central China Normal University, Wuhan, 430079 Hubei China

**Keywords:** MicroRNA-disease interaction, Heterogeneous omics data, Kernel neighborhood similarity, Bidirectional propagation, Diffusion component analysis

## Abstract

**Background:**

Studies have shown that miRNAs are functionally associated with the development of many human diseases, but the roles of miRNAs in diseases and their underlying molecular mechanisms have not been fully understood. The research on miRNA-disease interaction has received more and more attention. Compared with the complexity and high cost of biological experiments, computational methods can rapidly and efficiently predict the potential miRNA-disease interaction and can be used as a beneficial supplement to experimental methods.

**Results:**

In this paper, we proposed a novel computational model of kernel neighborhood similarity and multi-network bidirectional propagation (KNMBP) for miRNA-disease interaction prediction, especially for new miRNAs and new diseases. First, we integrated multiple data sources of diseases and miRNAs, respectively, to construct a novel disease semantic similarity network and miRNA functional similarity network. Secondly, based on the modified miRNA-disease interactions, we use the kernel neighborhood similarity algorithm to calculate the disease kernel neighborhood similarity and the miRNA kernel neighborhood similarity. Finally, we utilize bidirectional propagation algorithm to predict the miRNA-disease interaction scores based on the integrated disease similarity network and miRNA similarity network. As a result, the AUC value of 5-fold cross validation for all interactions by KNMBP is 0.93126 based on the commonly used dataset, and the AUC values for all interactions, for all miRNAs, for all disease is 0.93795、0.86363、0.86937 based on another dataset extracted by ourselves, which are higher than other state-of-the-art methods. In addition, our model has good parameter robustness. The case study further demonstrated the predictive performance of the model for novel miRNA-disease interactions.

**Conclusions:**

Our KNMBP algorithm efficiently integrates multiple omics data from miRNAs and diseases to stably and efficiently predict potential miRNA-disease interactions. It is anticipated that KNMBP would be a useful tool in biomedical research.

## Background

MicroRNAs (miRNAs) are a category of single-stranded small-non-coding RNAs(~ 22 nt) which play important roles in gene regression via interference in post-transcriptional regulation [1, 2]. In the past decades, microRNAs were found in eukaryotes and viruses besides prokaryotes [3]. Previous research has shown that miRNAs was related to several human diseases like cancer, Alzheimer’s disease and Diabetes Mellitus etc. [4–6]. miR-375 was found to be significant in the growth and response to metabolic stress of pancreatic islets [7].miR-21 negatively regulated Pdcd4 which can suppress TPA-induced neoplastic transformation [8]. miRNA-200 was detected in the metastasis of gastric adenocarcinoma cells [9]. miR-146a is a tumor suppressor inhibit NF-κB activity related to promotion and suppression of tumor growth [10].

Wang et al. [11] constructed a Directed Acyclic Graph (DAG) to describe a disease based on the MeSH descriptors. Then they calculated the disease semantic similarity by the DAG, and combined with the known miRNA-diseases interaction to construct the miRNA functional similarity, which was also used to preliminarily infer new potential functions or related diseases of miRNAs. Xu et al. [12] proposed a support vector machine (SVM) to predict the interaction between miRNA and tumor, but since the current database rarely provides a list of non–cancer miRNAs, therefore, the lack of negative samples leads to a supervised learning model that is not well suited for large-scale disease-miRNA interaction prediction.

The miRNA-disease interaction prediction problem can be regarded as a classification problem that lacks negative samples. According to this feature, a large number of network-based semi-supervised methods have been proposed, most of which are based on similar miRNAs (diseases) are more likely to interact with the same disease (miRNA). Chen et al. [13] adopted restart random walk (RWRMDA) to predict the potential miRNA-disease interaction, which restarted the known miRNA-disease interaction network, using random walks on miRNA functional similarity network to predict potential miRNA-disease interaction. Since the restart operator of RWRMDA is based on the known miRNA-disease interaction network, this method does not apply to predictions of new diseases that are not associated with any miRNA. The regularized least squares algorithm (RLSMDA) was also proposed by Chen et al. [14] in 2015 to predict miRNA-disease interactions, which uses both the disease semantic similarity and the miRNA functional similarity to calculate miRNA-disease interaction scores, and the weighted linear combination of the two scores was used as the final result. The method combined disease similarity network and miRNA similarity network to predict simultaneously, which improves the prediction accuracy and enhanced the predictive power of the model to some extent. However, the model is highly dependent on parameters, and how to set appropriate parameters is the defect of the model. Subsequently, in 2018, Chen et al. [15] released a Graph Regression model to predict miRNA–disease interactions by using singular value decomposition (SVD) to decompose the interaction matrix, the disease similarity matrix and the miRNA similarity matrix, then using partial least squares (PLS) to perform graph regression in interaction space, miRNA similarity space, and disease similarity space. SVD decomposition and PLS regression can eliminate noise to a certain extent, but it also causes information loss, which leads to the reduction of model accuracy. Recently, Chen et al. proposed two novel models: the hierarchical clustering recommendation algorithm [16] (BNPMDA) and the low rank matrix decomposition [17] (IMCMDA) algorithm to predict potential miRNA–disease interactions. Both models have the advantage of fewer parameters, but the former uses only known miRNA-disease interaction networks for inference, so it cannot predict new miRNAs and new diseases, and the latter leads to a reduction in prediction accuracy due to matrix decomposition. The miRNA functional similarity used in the above algorithms is based on the method of Wang et al. [11], which depends on the known miRNA-disease interactions, so these models cannot predict new miRNAs.

Luo et al. [18] proposed a Kronecker regularized least squares, which calculated miRNA functional similarity based on miRNA-gene interaction network and gene weight network, combined with disease semantic similarity to predict potential miRNA-disease interactions. The model enhances the predictive power of new miRNAs by integrating heterogeneous omics data of miRNAs, but the model is highly dependent on the weight coefficients of different similarity measurements, which greatly affects its promotion and practical application ability. Xiao et al. [19] constructed a graph regularized non-negative matrix factorization method, which decomposes the modified known miRNA-disease interaction network, and uses miRNA functional similarity and disease semantic similarity to construct regularization operators for prediction. The model can predict new miRNAs and new diseases, but more model parameters and stronger parameter dependencies also reduce the performance of the model. Both of these models use information outside the miRNA-disease interaction dataset to construct miRNA functional similarity, which enhances their ability to predict new miRNAs. However, they only use MeSH descriptors to describe disease similarity, resulting in a sparsely diseased network, which limits the predictive performance of the model.

Here, we propose a new framework, kernel neighborhood similarity and multi-network bidirectional propagation (KNMBP), which uses multiple omics data to infer unknown miRNA-disease interactions. KNMBP uses disease-gene interactions, disease-biological process interactions, and disease semantic information to construct a novel disease semantic similarity network, using miRNA-target interactions and gene weight networks to construct a novel miRNA functional similarity network. Different from previous methods, the miRNA functional similarity and disease semantic similarity calculated in this paper does not utilize the known miRNA-disease interaction, but excavates more feature information of miRNA and disease from other latest datasets, which greatly expands our ability to predict new miRNA and disease. The accumulated research [15, 20] shows that the known miRNA-disease interaction network also contains important feature information of miRNA and disease, and the reasonable use of this information can well enhance the prediction ability of the model. In these considerations, based on the modified miRNA-disease interaction, we use the kernel-based neighborhood similarity algorithm to calculate the disease kernel neighborhood similarity and miRNA kernel neighborhood similarity. Finally, based on the integrated miRNA (disease) similarity network, we constructed a bidirectional propagation model to predict potential miRNA-disease interaction scores. The experimental results show that KNMBP not only has a good ability to predict new interactions, new miRNAs and new diseases, but also has the advantage of parameter robustness.

## Methods

### Methods overview

**Fig. 1:**
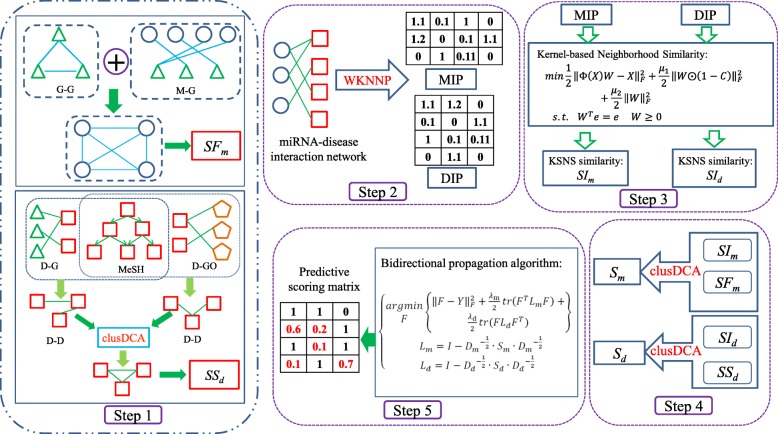
The flow diagram of KNMBP model. In Step 1 and Step 2, the red box indicates disease, the grass green triangle indicates the gene, the circle indicates the miRNA, the pentagon indicates the biological process corresponding to the disease, *SF*_*m*_ and *SS*_*d*_ represent improved miRNA functional similarity and disease semantic similarity, respectively, WKNNP represents a weighted k-neighborhood profile algorithm used to preprocess the interaction matrix. In Step 3, *SI*_*m*_ and *SI*_*d*_ represent disease kernel neighborhood similarity and miRNA kernel neighborhood similarity, respectively. In Step 4, clusDCA represents the network fusion algorithm based on diffusion component analysis

To predict unknown miRNA-disease interactions, we propose a new KNMBP model with five parts, as shown in Fig. 1. First, we calculate miRNA functional similarity and disease semantic similarity by using multiple histological data other than miRNA-disease interaction information (as shown in step 1 of Fig. 1). Second, based on the modified known miRNA-disease interaction network, we use the kernel-based neighborhood similarity model (KSNS) to calculate the disease kernel neighborhood similarity and miRNA kernel neighborhood similarity (as shown in step 2 and step 3 of Fig. 1). Finally, based on the integrated miRNA (disease) similar network calculated by Diffusion Component Analysis (clusDCA), we released a bidirectional propagation algorithm to predict unknown miRNA-disease interaction scores (as shown in step 4 and step 5 in Fig. 1).

### Dataset collection

In order to fairly compare the performance of the model, we used two benchmark datasets to conduct experiments.

For benchmark dataset I, we utilized the dataset of miRNA-disease interaction prediction established by Chen et al. [16, 17]. The dataset I consists of three parts: First, 5430 interactions between 383 diseases and 495 miRNAs were extracted from HMDD v2.0 [21]. Second, based on the Medical Subject Headings (MeSH) descriptors in the U.S. National Library of Medicine, two semantic similarity matrices of diseases were established by wang et al. [11] and Xuan et al. [22], respectively. Third, the functional similarity matrix of miRNA was established by Lu et al. [23]. All these data can be downloaded from https://github.com/IMCMDAsourcecode/IMCMDA. However, Dataset I is based on the old version (HMDD v2.0), and it also has the disadvantage that the disease semantic similarity is very sparse and the miRNA functional similarity depends on the known miRNA-disease interaction. Therefore, we extracted information about miRNAs and diseases from several latest databases and built benchmark dataset II. We describe the establishment of dataset II from three aspects.

First, extract information about the disease. The Comparative Toxicogenomics Database (CTD) is an important database of disease research that provides a wealth of interactive information between disease and chemistry, genetic products, phenotypes and the environment [24]. Disease items in CTD are described by MeSH ID, which is a hierarchical vocabulary that provides a strict classification system for studying the relationships among various diseases, and the relationships between any diseases can be illustrated by a directed acyclic graph (DAG). For example, the MeSH ID of the disease “Deletion Syndrome (Partial)” was “MesH:C538288” in CTD, whose parent diseases are “Chromosome Deletion” and “Chromosome Disorders”, and the corresponding MesH ID were “MesH:D002872” and “MesH: D025063”, respectively. In order to get a detailed description of the disease, we download 12,988 diseases, including the names of diseases, multiple ID representations of the diseases, and information about their parent nodes. Furthermore, we downloaded gene-disease interactions, including 25,114,553 interactions between 46,045 genes and 7163 diseases. At the same time, disease-GO biological process interactions, including 1,727,119 interactions between 13,126 GOs and 7116 diseases were also downloaded.

Second, extract information about the miRNA. In order to accurately describe the relationship between miRNAs, we extracted as complete as possible miRNA interaction information from multiple latest databases. We obtained the miRNA-gene interaction information from experimentally verified databases, including TarBase (version 8.0) [25], miRTarBase (version 7.0) [26], miRNAMAP (version 2.0) [27], miRecord (version 4) [28]. DIANA-TarBase v8 is a reference database for indexing experimentally supported microRNA targets, has more than a decade of support in the field of non-coding RNA [25]. We downloaded 927,119 miRNA-gene interactions from the database, after the removal of non-human gene and converted the gene ID into Entrez Gene identifiers, a total of 423,392 interactions between 18,345 genes and 1084 miRNAs are retained. Meanwhile, we performed ID transformation of the genes in the miRTarBase database, deleted the null miRNAs and target genes, and finally obtained 381,088 interactions between 2599 miRNAs and 15,064 genes. Similarly, we extracted 83,071 interactions between 1135 target genes and 471 miRNAs from miRNAMAP, and obtained 1269 interactions between 767 target genes and 203 miRNAs from the miRecord. Based on miRBase [29], all of the above miRNAs were transformed into the v22 version using the R package ‘miRBaseConverter’, and the null and duplicate miRNAs were deleted. After integration, a total of 588,134 interactions between 2814 miRNAs and 18,468 genes were obtained. In addition, Lee et al. [30] integrated 21 omics data from multiple organisms by modifying bayes and used logarithmic likelihood scores to measure the probability of interaction between two genes with true functional links. To build similarity networks of genes, we downloaded the human weighted gene network data from the HumanNet database, which contained the log likelihood score of 476,399 interactions among 16,243 genes.

Third, extract interactive information of miRNA and disease. The human microRNA Disease Database (HMDD) collects large amounts of human miRNA-disease interactions from genetics, epigenetics, circulating miRNA and miRNA target interactions, and provides detailed annotation of miRNA-disease interactions [21]. In June 28, 2018, HMDD (version 3.0) [31] was also released, which provides 200.2% of human miRNA-disease interactions and has more evidence to classify. We extracted the disease information with MeSH ID or OMIM ID from HMDD v3.0, removed duplicate miRNA-disease interactions, and obtained 14,457 interactions between 1045 miRNAs and 627 diseases. To ensure all the miRNA similarity and all the disease similarity can be calculated, we delete the diseases and miRNAs not in the above two datasets, and finally got 10,561 interactions between 574 miRNAs and 579 diseases. The details of the two benchmark datasets are shown in Additional file 1.

### Construction of disease semantic similarity network

In fact, most methods use MeSH descriptors to construct a directed acyclic graph of the disease, which contains common information between different diseases is used to describe the disease similarity, which leads to a sparsely similar network [16, 17]. In order to construct a more reasonable disease semantic similarity, we make full use of the various omics data to calculate the similarity of the disease. Protein-encoding genes can affect the pathogenesis of the disease to some extent [32], so disease-gene interactions also imply some features of the disease. Similarly, the gene ontology biological process of the disease is also the reflection of some characteristics of the disease. In this paper, we combine the disease-gene interactions (D-G) and disease-GO biological process interactions datasets (D-GO), and the MeSH descriptors of the disease, using the MultiSourcDSim model proposed by Lei et al. [33] to calculate the disease semantic similarity.

Based on the MeSH descriptor, a directed acyclic graph (DAG) can be used to describe the semantic relationship between diseases. Any disease d in the DAG can be expressed as *DAG*(*d*) = (*d*, *S*(*d*), *F*(*d*), *A*(*d*)), where *S*(*d*) and *F*(*d*), representing the set of direct child nodes and direct parent nodes of disease *d*, respectively, and *A*(*d*) represents the set constituted by all ancestor nodes of disease *d*.

First, combining the disease interaction dataset (D-G or D-GO) and DAG, the frequency *FT*_*c*_(*d*) of any disease d in the DAG can be calculated:

1 $$ {FT}_c(d)={f}_c(d)+\sum \limits_{d\in S(d)}{FT}_c(d) $$

where *f*_*c*_(*d*) represents the frequency of d in the interaction dataset c, it can be seen that the occurrence frequency of d in DAG is equal to the sum of the occurrence frequency of all its direct child nodes and the frequency of itself in the interaction dataset. Then, normalize the frequency of disease occurrence as follow:

2 $$ {PT}_c(d)=\frac{PT_c(d)}{PT_c(root)} $$

Where, *PT*_*c*_(*root*) represents the occurrence frequency of the root node in DAG. According to Eqs. 1 and 2, it can be known that 0 ≤ *PT*_*c*_(*t*) ≤ 1. Based on the more information shared, the higher the similarity. The disease similarity can be obtained:

3 $$ {S}_c\left({d}_1,{d}_2\right)={\displaystyle \begin{array}{c}\mathit{\operatorname{MAX}}\\ {}d\in COM\left({d}_1,{d}_2\right)\end{array}}\left(\frac{2\times \mathit{\log}\left({PT}_c(d)\right)}{\mathit{\log}\left({PT}_c\left({d}_1\right)\right)+\mathit{\log}\left({PT}_c\left({d}_2\right)\right)}\right) $$

Where, *COM*(*d*_1_, *d*_2_) is the set of the minimum common ancestor of the disease *d*_1_ and *d*_2_, and it is easy to see that 0 ≤ S_*c*_(*d*_1_, *d*_2_) ≤ 1. According to D-G and D-GO, we can obtain two disease similarity networks {*S*_*c*_, *c* = 1, 2}. After that, the clusDCA [34] was used to integrate the disease similar networks, and the integrated semantic similar network *SS*_*d*_ was finally obtained.

### Construction of miRNA functional similarity network

In order to overcome the dependence of miRNA functional similarity on known miRNA-disease interaction network, the algorithm can predict miRNAs not associated with any disease. We calculate the miRNA functional similarity by means of Luo [18] and Xiao’s [19] methods. Specifically, we used miRNA target gene interaction network and gene similarity network to calculate miRNA similarity.

First, we normalized and symmetrized the log-likelihood score data between genes downloaded from HumanNet:

4 $$ {S}^g\left({g}_i,{g}_j\right)=\Big\{{\displaystyle \begin{array}{c}\frac{LLS\left(i,j\right)}{{\operatorname{MAX}}_{LLS}},\kern2em LLS\left(i,j\right)\ne 0\\ {}\frac{LLS\left(j,i\right)}{{\operatorname{MAX}}_{LLS}},\kern2em LLS\left(i,j\right)=0 andLLS\left(j,i\right)\ne 0\\ {}0,\kern5.00em Otherwise\end{array}}\operatorname{} $$

Where *S*^*g*^(*g*_*i*_, *g*_*j*_) represents the similarity between gene *g*_*i*_ and gene *g*_*j*_, *LLS*(*i*, *j*) represents the log-likelihood score between gene *g*_*i*_ and gene *g*_*j*_, *MAX*_*LLS*_ represents the maximum log-likelihood score. At this point, we can define the similarity between any gene *g*_*i*_ and any gene set G:

5 $$ {S}^g\left({g}_i,\mathrm{G}\right)=\underset{g_j\in \mathrm{G}}{\max}\left\{{S}^g\left({g}_i,{g}_j\right)\right\} $$

Where, *S*^*g*^(*g*_*i*_, G) represents the similarity between *g*_*i*_ and G. Then, we can get the functional similarity between miRNA *m*_*i*_ and miRNA *m*_*j*_:

6 $$ {SF}_m\left({m}_i,{m}_j\right)=\frac{\sum_{g\in {G}_i}{S}^g\left(g,{G}_i\right)+{\sum}_{g\in {G}_j}{S}^g\left(g,{G}_j\right)}{\left|{G}_i\right|+\left|{G}_j\right|} $$

Where, *SF*_*m*_(*m*_*i*_, *m*_*j*_) represents the functional similarity between *m*_*i*_ and *m*_*j*_, *G*_*i*_ represent the gene set associated with *m*_*i*_, and |*G*_*i*_| represent the number of genes in the set *G*_*i*_.

### Kernel-based neighborhood similarity

Reasonable use of known miRNA-disease interaction information can greatly improve the performance of the model [17, 18]. In this paper, based on the known miRNA-disease interactions, we used the kernel-based neighborhood similarity (KSNS) [35] to calculate miRNA (disease) kernel neighborhood similarity. KSNS not only comprehensively utilizes the distance similarity and structural similarity of samples, but also fully excavates the nonlinear structural similarity information between samples, achieving a good prediction effect in lncRNA-protein interaction prediction. In addition, to overcome the sparse problem of the interaction matrix, a weighted k-neighborhood profile (WKNNP) algorithm was proposed by Xiao et al. [19] to preprocess the interaction matrix, achieved good results. Based on the above two points, we first use WKNNP to preprocess the known interaction matrix, and then uses KSNS to calculate the kernel neighborhood similarity of miRNA (disease).

Let the matrix X of the NM rows and ND columns represent the miRNA-disease interaction matrix, then X can be expressed as: $\mathrm{X}=\left[{M}_1^T,{M}_2^T,\cdots, {M}_{NM}^T\right]=\left[{D}_1,{D}_2,\cdots, {D}_{ND}\right]$, where *M*_*i*_ is the *i*th row vector of X, could be regarded as the interaction profile feature of miRNA *m*_*i*_; *D*_*j*_ is the *j*th column vector of X, could be regarded as the interaction profile feature of disease *d*_*j*_.

According to the WKNNP algorithm, we make use of K-nearest neighbor feature of *m*_*i*_ to enrich the interaction profile *M*_*i*_, then the modified interaction profile ${\hat{M}}_i$ of *m*_*i*_ is as follows:

7 $$ {\hat{M}}_i=\frac{1}{Q_{m_i}}{\sum}_{k=1}^K{w}^k{M}_k $$

Where ${Q}_{m_i}={\sum}_{m_{j\in N\left({m}_i\right)}}{SF}_m\left({m}_i,{m}_j\right)$ denotes regularization weight, and *N*(*m*_*i*_) represents the K nearest set of *m*_*i*_ (For sake of simplicity, let K = 15 in the paper). *w*^*k*^ is the weight coefficient of the *k*th neighbor, and decay factor *α* ∈ [0, 1] (For sake of simplicity, let *α* = 0.8 in the paper), It is easy to see that the more closer miRNAs have higher weight coefficients. At this point, the modified interaction profile matrix can ${X}_M=\left[{\hat{M}}_1^T,{\hat{M}}_2^T,\cdots, {\hat{M}}_{NM}^T\right]$ be obtained through Eq. 7. Similarly, we can get the disease modified interaction profile matrix ${X}_d=\left[{\hat{D}}_1,{\hat{D}}_2,\cdots, {\hat{D}}_{ND}\right]$. Finally, the modified interaction profile matrix X is shown as follows:

8 $$ \hat{X}=\max \left\{X,\frac{1}{2}\left({X}_m+{X}_d\right)\right\} $$

Now, based on the $\hat{\mathrm{X}}$, we make use of KSNS to calculate miRNA (disease) kernel neighborhood similarity. First, we construct the K-neighboring discriminant matrix of miRNA based on the miRNA functional similarity:

9 $$ {C}_{i,j}=\Big\{{\displaystyle \begin{array}{c}1,\kern2em j\in N\left({m}_i\right)\\ {}0,\kern2em j\notin N\left({m}_i\right) ori=j\end{array}}\operatorname{} $$

Where *N*(*m*_*i*_) represents the set of NK nearest miRNAs of *m*_*i*_, NK = ⌊*PN* × *N*⌋, PN denotes neighbors proportion parameter, *N* is the total number of samples, ⌊∙⌋ means round down. Then weight matrix *W* of miRNA is as follow:

10 $$ {\displaystyle \begin{array}{c}\mathit{\min}\frac{1}{2}{\left\Vert \Phi (X)W-\Phi (X)\right\Vert}_F^2+\frac{\mu_1}{2}{\left\Vert W\bigodot \left(1-C\right)\right\Vert}_F^2+\frac{\mu_2}{2}{\left\Vert W\right\Vert}_F^2\\ {}s.t.{W}^Te=e\ W\ge 0\ \mathit{\operatorname{diag}}(W)=0\end{array}} $$

Where, Φ(∙) denotes kernel function, ‖∙‖_*F*_ representsFrobenius norm, ⨀ is an element-by-element multiplication, *μ*_1_ is non-neighborhood control parameters, *μ*_2_ is similarity regularization parameters, *e* = (1, 1, ……, 1)^*T*^. The first item of constraint requires the sum of reconstruction weights of each sample to be 1, the second requires that all elements in W are non-negative, and the third term indicates that the self-similarity of miRNA is 0. Using the Lagrange multiplier method and the Karush-Kuhn-Tucker (KKT) condition, the iterative formula of W is as follows:

11 $$ {W}_{ij}=\frac{{\left[k\left(X,\mathrm{X}\right)+{\mu}_1W\bigodot C\right]}_{ij}}{{\left[k\left(X,\mathrm{X}\right)W+{\mu}_1W+{\mu}_2W\right]}_{ij}}{W}_{ij} $$

Where *k*(*X*, X) represents the kernel matrix of X. In this paper, we select Gaussian kernel function, which is represented as:

12 $$ k\left({x}_i,{x}_j\right)=\left\langle \Phi \left({x}_i\right),\Phi \left({x}_j\right)\right\rangle =\exp \left(-{\left\Vert {x}_i-{x}_j\right\Vert}^2/\upgamma \right) $$

Where *k*(*x*_*i*_, *x*_*j*_) is the kernel of any two samples of *x*_*i*_, *x*_*j*_. $\upgamma =\frac{\sum {\left\Vert {x}_i\right\Vert}^2}{NM}$ represents the regularized bandwidth parameter. After that, we conducted multiple normalization operations on the weight matrix *W* to obtain the miRNA kernel neighborhood similarity matrix *SI*_*m*_, and the normalization formula is as follows:

13 $$ {SI}_m={D}^{-\frac{1}{2}}{\mathrm{W}}^T{D}^{-\frac{1}{2}} $$

Where, the diagonal matrix D =  *diag* (*d*_1_, *d*_2_, …, *d*_*NM*_), ${d}_j=\sum \limits_{i=1}^{NM}{W}_{i,j}$. Similarly, we can get the disease kernel neighborhood similarity *SI*_*d*_. Then the clusDCA [34] was used to integrate the miRNA functional similarity *SF*_*m*_ (disease semantic similarity matrix *SS*_*d*_) and kernel neighborhood similarity *SI*_*m*_ (kernel neighborhood similarity *SI*_*d*_) to obtain the final miRNA similarity matrix *S*_*m*_= (disease similarity matrix *S*_*d*_).

### Bidirectional propagation algorithm

Based on miRNA similarity, disease similarity and known miRNA-disease interaction information, we proposed a bidirectional propagation algorithm to predict the miRNA-disease interaction score.

Let (*F*)_*NM* × *ND*_ be the miRNA-disease interaction score matrix, then *F* can be decomposed as $F=\left[{FM}_1^T,{FM}_2^T,\cdots, {FM}_{NM}^T\right]=\left[{FD}_1,{FD}_2,\cdots, {FD}_{ND}\right]$, Where, ${FM}_i^T$ represents the predicted interaction score of miRNA *m*_*i*_ with all diseases, and *FD*_*j*_ denotes the predicted interaction score of disease *d*_*j*_. Based on the hypothesis that higher similarity miRNAs are more likely to be interacted with the same disease, we can get:

14 $$ \sum \limits_{i,j}^M{s}_{i,j}^m{\left\Vert \frac{1}{\sqrt{d_i^m}},{FM}_i,-,\frac{1}{\sqrt{d_j^m}},{FM}_j\right\Vert}^2= tr\left({F}^T\left(I-{D_m}^{-\frac{1}{2}}\bullet {S}_m\bullet {D_m}^{-\frac{1}{2}}\right)F\right) $$

Where ${s}_{i,j}^m={\left({S}_m\right)}_{i,j}$ denotes the similarity of *m*_*i*_ and *m*_*j*_. ${d}_i^m=\sum \limits_{j=1}^{NM}{s}_{i,j}^m$, and the diagonal matrix ${D}_m=\mathit{\operatorname{diag}}\left({d}_1^{\mathrm{m}},{d}_2^{\mathrm{m}},\cdots, {d}_{NM}^{\mathrm{m}}\right)$. Similarly for diseases, we can get:

15 $$ \sum \limits_{u,v}^{ND}{s}_{u,v}^d{\left\Vert \frac{1}{\sqrt{d_u^d}}{FD}_s-\frac{1}{\sqrt{d_v^d}}{FD}_t\right\Vert}^2= tr\left({F}^T\left(I-{D_d}^{-\frac{1}{2}}\bullet {S}_D\bullet {D_d}^{-\frac{1}{2}}\right)F\right) $$

Where ${s}_{u,v}^d={\left({S}_d\right)}_{u,v}$ denotes the similarity of *d*_*u*_ and *d*_*v*_. ${d}_u^d=\sum \limits_{k=1}^{ND}{s}_{u,k}^d$, and the diagonal matrix ${D}_d=\mathit{\operatorname{diag}}\left({d}_1^d,{d}_2^d,\cdots, {d}_{ND}^d\right)$. By this stage, the bidirectional propagation algorithm can be obtained as follows:

16 $$ \Big\{{\displaystyle \begin{array}{c}\begin{array}{c} argmin\\ {}F\end{array}\left\{{\left\Vert F-Y\right\Vert}_F^2+\frac{\lambda_{\mathrm{m}}}{2} tr\left({F}^T{L}_mF\right)+\frac{\lambda_{\mathrm{d}}}{2} tr\left({FL}_d{F}^T\right)\right\}\\ {}\kern0.50em {L}_m=I-{D_m}^{-\frac{1}{2}}\bullet {S}_m\bullet {D_m}^{-\frac{1}{2}}\\ {}{L}_d=I-{D_d}^{-\frac{1}{2}}\bullet {S}_D\bullet {D_d}^{-\frac{1}{2}}\end{array}}\operatorname{} $$

Where ${\left\Vert F-Y\right\Vert}_F^2$ represents the overall prediction error, which is required to be as small as possible, *λ*_m_ and *λ*_d_ are the Laplacian regularization parameters of miRNA and disease, respectively. The derivative of Eq. 16 for F is as follows:

17 $$ \frac{\partial Q(F)}{F}=2\left(\mathrm{F}-\mathrm{Y}\right)+{\lambda}_m{L}_mF+{\lambda}_d{FL}_d $$

In order to speed up the optimization of the gradient algorithm, we use AdaGrad algorithm [34] to adaptively choose the gradient step size. The details of the optimization algorithm to the proposed bidirectional propagation model are described in Algorithm 1. 
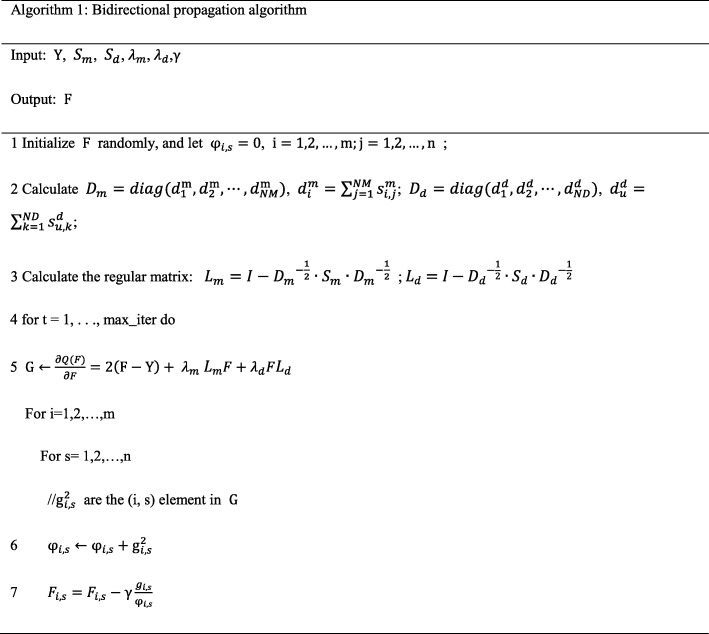


## Results

### Comparison with other methods

#### Experimental settings

To evaluate the performance of the KNMBP algorithm fairly, we performed the 5-fold cross-validation (*CV*) on Dataset I and Dataset II, and compared with the following methods: IMCMDA [17], BNPMDA [16] and RLSMDA [14], KRLSM [18], RWRMDA [13]. Specifically, for each method, we performed *CV* four times, each time using a different seed, and the mean value of the AUC values under different seeds was taken as the final AUC value of the method. The miRNA-disease interaction matrix Y ∈ *R*^*NM* × *ND*^ had *NM* rows for miRNAs and *ND* columns for diseases. We carried out three types of *CV* as follows [36]:

1. CV_a_ : CV on all miRNA-disease pairs. In order to ensure that the known interactions could be evenly distributed, we randomly divided the known and unknown interactions into five equal parts, one of which was selected as the test set in turn, and the association contained in it was deleted as the training set.
2. CV_m_ : CV on miRNAs (row vectors in Y), all miRNAs were randomly divided into five equal parts, one of which was selected as the test set in turn, and its association was deleted as the training set.
3. CV_d_ : CV on diseases (column vectors in Y), all diseases were randomly divided into five equal parts, one of which was selected as the test set in turn, and its association was deleted as the training set.

In each crossover experiment, Under *CV*_*a*_, 80% of Y elements are used as the training set, and the remaining 20% are test set; Under *CV*_*m*_, 80% of rows in Y are used as the training set, and the remaining 20% are test set; Under *CV*_*d*_, 80% of columns in Y are used as the training set, and the remaining 20% are test set. In Dataset I, since the disease semantic similarity matrix is sparse, and the miRNA functional similarity relies on known miRNA-disease interactions, most of the methods only perform *CV*_*a*_ experiment. Therefore, we only perform *CV*_*a*_ on Dataset I, and perform the above three *CV* on Dataset II.

In this paper, we use the grid method to find the optimal combination of parameters. For KNMBP, the parameters are as follows: neighbors proportion parameter PN was selected from {10%, 30%, 50%, 70%, 90%}; non-neighborhood control parameters *μ*_1_ and similarity regularization parameters *μ*_2_ were selected from { 2^0^, 2^1^, 2^2^, 2^3^, 2^4^ }; For Laplace regularization parameters *λ*_m_ and *λ*_*d*_, we set *λ*_m_ = *λ*_d_ and choose the two parameters from { 2^−2^, 2^−1^, 2^0^, 2^−1^, 2^−2^ }. For RWRMDA, {0, 0.1, ⋯, 0.9} for restart probability *r* and {1, 2, 3, ⋯, 6} for walk times; For KRLSM, with the authors’ recommendations, we set *σ* = 1, the weight parameters were selected from {0, 0.1, ⋯, 1};For RLSMDA, weight parameters *w* = 0.5 , the regularization parameters *η*_*m*_ = *η*_*d*_ and were selected from {0, 0.1, ⋯, 1}; For IMCMDA, the subspace dimension *r* was selected from {50, 100, ⋯, 500}.

#### Cross validation

For each *CV*, we calculated the prediction interaction scores of the test set by the above six methods, and normalized all the prediction interaction scores as follows:

$$ \hat{PS}\left(i,j\right)=\frac{PS\left(i,j\right)-\mathit{\min} PS}{maxPS- minPS} $$

**Fig. 2:**
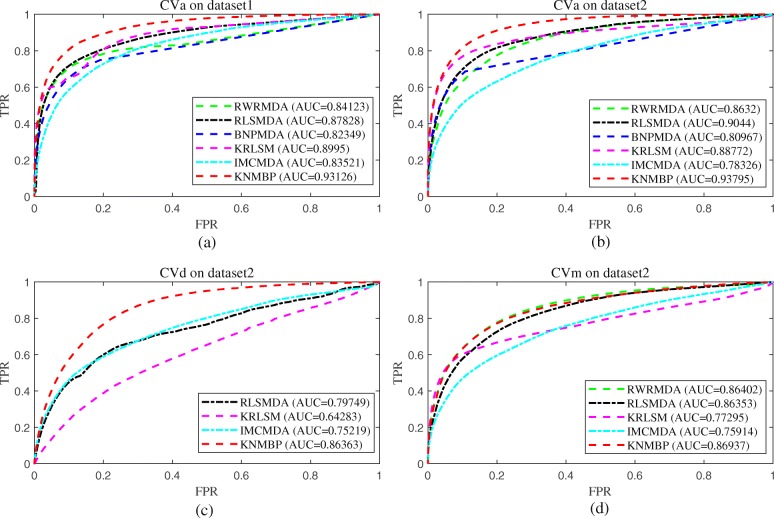
Performance comparisons between KNMBP and other state-of-the-art methods (RWRMDA, RLSMDA, BNPMDA, KRLSM, IMCMDA) in terms of AUC based on 5-fold cross validation. **a** perform CV_a_ on Dataset I; **b** perform CV_a_ on Dataset II; **c** perform CV_d_ on Dataset II; **d** perform CV_m_ on Dataset II

Where *PS*(*i*, *j*) represents the predicted interaction score of miRNA *m*_*i*_ and disease *d*_*j*_, *minPS* represents the minimum value of *PS*, and *maxPS* represents the maximum value of *PS*. Then, the [0,1] interval is equally divided into 1000, and each of the points is sequentially selected as a threshold, and calculate the True Positive Rate (TPR, sensitivity) and False Positive Rate (FPR, 1-specificity) under each specific threshold. After that, we calculate the mean value of the TPR and the FPR for each threshold under *CV*, draw the corresponding TPR and FPR curve. Figure 2 shows the optimal AUC and corresponding ROC curves for each model under *CV*. The optimal parameters of KNMBP and the corresponding AUC values are shown in Additional file 2.

In the above experiment, *CV*_*a*_ tested the predictive performance of the model for new interactions, and *CV*_*m*_ and *CV*_*d*_ tested the predictive performance for new miRNAs and new diseases, respectively. It can be seen that our method (KNMBP) achieves the best prediction results in Fig. 2. Specifically, based on Dataset I, the AUC value of KNMBP for *CV*_*a*_ can reach 0.93126, which is 9.67, 5.69, 11.57, 3.41, and 10.31% higher than RWRMDA, RLSMDA, BNPMDA, KRLSM, and IMCMDA, respectively. Based on Dataset II, the AUC value of KNMBP for CVa can reach 0.93795, which is 7.97, 3.58, 13.68, 5.31 and 16.49% higher than the other five methods respectively. Since BNPMDA based on binary recommendation algorithm needs to utilize known miRNA-disease interactions to achieve resource allocation, it cannot predict new miRNA and new diseases [16]. RWRMDA, which restarts the random walk on MiRNA similarity network, is also not suitable for prediction of new diseases [13]. Therefore, RLSMDA, KRLSM and IMCMDA were selected as comparison algorithms under *CV*_*d*_, and the AUC value of KNMBP could reach 0.86363, which was 7.66, 25.577 and 12.93% higher than the other three methods (RLSMDA, KRLSM, IMCMDA). For *CV*_*m*_, the AUC of KNMBP can reach 0.86937, which is 0.62, 0.67, 11.09, 5.31 and 12.68% higher than the other four methods (RWRMDA, RLSMDA, KRLSM, IMCMDA), respectively.

#### Parametric sensitivity analysis

In machine learning, with the change of experimental scenarios, the optimal parameter combination may be very different, and the parameter selection may have a huge impact on the performance of the model, so the sensitivity analysis of parameters is often very important. In this section, we focus on the influence of four parameters, namely, neighbor proportion parameter PN, Laplace regularization parameter *λ* = *λ*_m_ = *λ*_d_, non-neighborhood control parameter *μ*_1_ and similarity regularization parameter *μ*_2_, on the prediction performance of the model. Let F_*cv* = *c*_(*PN* = *i*, *λ* = *j*, *μ*_1_ = *s*, *μ*_2_ = *t*) represent the AUC value of the KNMBP algorithm when *cv* = *c*, *c* ∈ {1, 2, 3, 4} is performed and the parameters are set to *PN* = *i*, *λ* = *j*, *μ*_1_ = *s*, *μ*_2_ = *t*. In order to facilitate the visualization of the results, for each type of *CV* we combined the above four parameters in pairs to analyze the influence of the paired parameters on the predicted results of the model.

First, we consider the influence of neighbor proportion parameter PN and Laplace regularization parameter *λ* on the predictive performance of the model. When *PN* = *i*, *λ* = *j*, and the other two parameters change arbitrarily, we calculate the maximum AUC value of KNMBP (${\mathrm{maxAUC}}_{i,j}^c$), the average AUC value (${\mathrm{meanAUC}}_{i,j}^c$) and the minimum AUC value (${\mathrm{minAUC}}_{i,j}^c$), as shown below:

18 $$ {\displaystyle \begin{array}{c}{\mathrm{maxAUC}}_{i,j}^c=\max \left\{{F}_{cv=c}\left( PN=i,\lambda =j,{\mu}_1,{\mu}_2\right)\right|{\mu}_1\in \forall, {\mu}_2\in \forall \Big\}\\ {}{\mathrm{meanAUC}}_{i,j}^c=\mathrm{mean}\left\{{F}_{cv=c}\left( PN=i,\lambda =j,{\mu}_1,{\mu}_2\right)\right|{\mu}_1\in \forall, {\mu}_2\in \forall \Big\}\\ {}{\mathrm{minAUC}}_{i,j}^c=\min \left\{{F}_{cv=c}\left( PN=i,\lambda =j,{\mu}_1,{\mu}_2\right)\right|{\mu}_1\in \forall, {\mu}_2\in \forall \Big\}\end{array}} $$

**Fig. 3:**
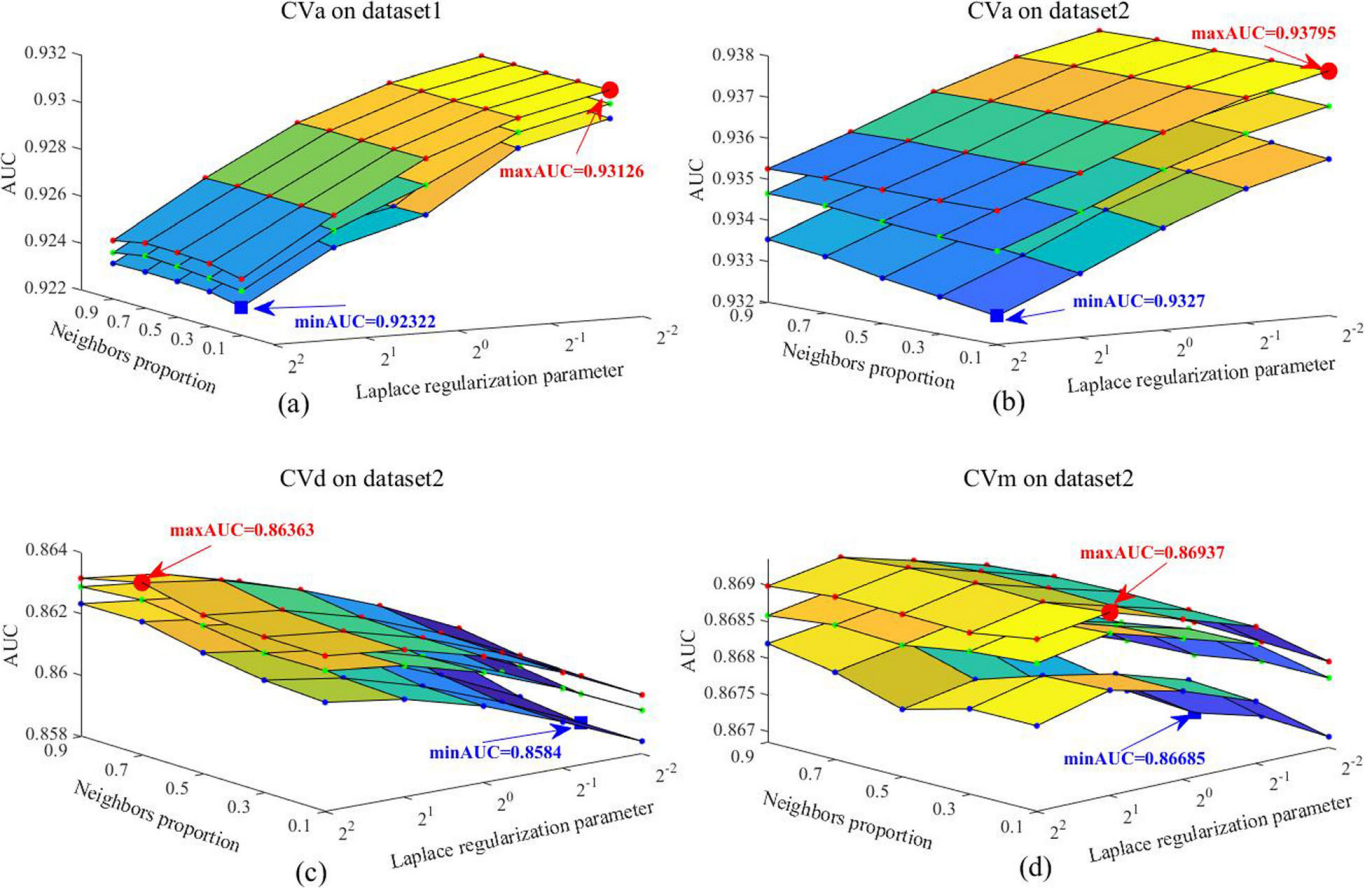
The influence of neighbor proportion parameter *PN* and Laplace regularization parameter *λ* on the predictive performance of the model. **a** CVa on dataset1; **b** CVa on dataset2; **c** CVd on dataset2; **d** CVm on dataset2

Where *μ*_1_ ∈ ∀ and *μ*_2_ ∈ ∀ represent arbitrary values of the parameters *μ*_1_ and *μ*_2_ within their range (*μ*_1_ , *μ*_2_∈ { 2^0^, 2^1^, 2^2^, 2^3^, 2^4^ }). When *cv* = 1, it means we perform *CV*_*a*_ on Dataset I; *cv* = 2 means we perform *CV*_*a*_ on Dataset II; *cv* = 3 means we perform *CV*_*d*_ on Dataset II; *cv* = 4 means we perform *CV*_*m*_ on Dataset II. In particular, under a certain *CV*, for every set of values of *PN* and *λ*, we first calculate the AUC values when *μ*_1_ and *μ*_2_ are arbitrarily changed within their range, then calculate the maximum, average and minimum values of this group of AUC values according to (20), and the results are shown in Fig. 3.

It can be seen from Fig. 3 that with the change of neighbor proportional parameter PN and Laplace regularization parameter *λ*, the AUC value of the model has a trend fluctuation, but the overall fluctuation range is small. Specifically, as shown in (a) of Fig. 3, the minAUC is 0.92322 when PN = 0.1 and *λ* = 4, and the maxAUC is 0.93126 when *PN* = 0.1 and *λ* = 1/4, with an overall relative change of 0.87%. Similarly, in (b), (c), and (d) of Fig. 3, the relative ranges of overall AUC changes with respect to the model caused by *PN* or *λ* are 0.56, 0.61, and 0.29%, respectively. The result shows that KNMBP has strong stability related to neighbor proportional parameter *PN* and Laplace regularization parameter *λ*.

Now we consider the non-neighborhood control parameter *μ*_1_ and similarity regularization parameter *μ*_2_. Similarly, When *μ*_1_ = *s*, *μ*_2_ = *t*, the other two parameters change arbitrarily, we calculate the maximum AUC value of KNMBP (${\mathrm{maxAUC}}_{s,t}^c$), the average AUC value (${\mathrm{meanAUC}}_{s,t}^c$) and the minimum AUC value (${\mathrm{minAUC}}_{s,t}^c$), as shown below:

19 $$ {\displaystyle \begin{array}{c}{\mathrm{maxAUC}}_{s,t}^c=\max \left\{{F}_{cv=c}\left( PN,\lambda, {\mu}_1=s,{\mu}_2=t\right)\right| PN\in \forall, \lambda \in \forall \Big\}\\ {}{\mathrm{meanAUC}}_{s,t}^c=\mathrm{mean}\left\{{F}_{cv=c}\left( PN,\lambda, {\mu}_1=s,{\mu}_2=t\right)\right| PN\in \forall, \lambda \in \forall \Big\}\\ {}{\mathrm{minAUC}}_{s,t}^c=\min \left\{{F}_{cv=c}\left( PN,\lambda, {\mu}_1=s,{\mu}_2=t\right)\right| PN\in \forall, \lambda \in \forall \Big\}\end{array}} $$

Where *PN* ∈ ∀ and *λ* ∈ ∀ represent arbitrary values of the parameters *PN* and *λ* within their range (*PN* ∈ {10%, 30%, 50%, 70%, 90%} , *λ*∈ { 2^−2^, 2^−1^, 2^0^, 2^−1^, 2^−2^ }). Then the effect of these two parameters on the prediction performance of the model is shown in Additional file 3. As can be seen from (a), (b), (c) and (d) in Additional file 3, when the parameters *μ*_1_ and *μ*_2_ change in a certain range, the maxAUC value, meanAUC value and minAUC value of the model are almost flat, indicating that these two parameters have little influence on the prediction performance of the model. According to Fig. 3 and Additional file 3, when the parameters of the model change within a certain range, KNMBP can always achieve better prediction performance, indicating that our algorithm has strong parameters robustness.

### Case study

**Fig. 4:**
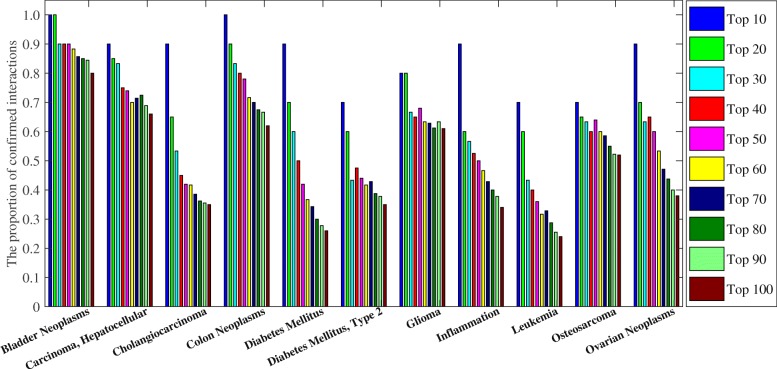
For different thresholds, the proportion of candidate mirnas that have been confirmed to be associated with the disease

To further demonstrate the predictive performance of KNMBP algorithm for novel miRNA-disease interactions, experiments were performed on the older version of HMDD (v2.0, June 20, 2013), and the prediction results were validated with the newer version of HMDD (v3.0, June 28, 2018). We downloaded the miRNA-disease interactions from HMDD v2.0 and extracted the disease data with MeSH ID or OMIM ID according to the details of the disease provided by HMDD v3.0. After processing, we obtained 2157 interactions of 166 diseases and 299 miRNAs, and constructed semantic similarity scores of these diseases and functional similarity scores of these miRNAs according to (2.2.1) and (2.2.2). The KNMBP was used for prediction, and the candidate miRNAs of 166 diseases ranked according to their predicted scores were provided in Additional file 4. Figure 4 shows the confirmed ratio of candidate miRNAs for 11 diseases under different thresholds. For example, the top 10 predicted scores of candidate miRNAs for Bladder Neoplasms are all confirmed in HMDD v3.0. Twenty-seven of the top 30 predicted scores were confirmed in HMDD v3.0. As can be seen from Fig. 4, most of the top candidate miRNAs for these diseases can be confirmed in the latest version.

**Fig. 5:**
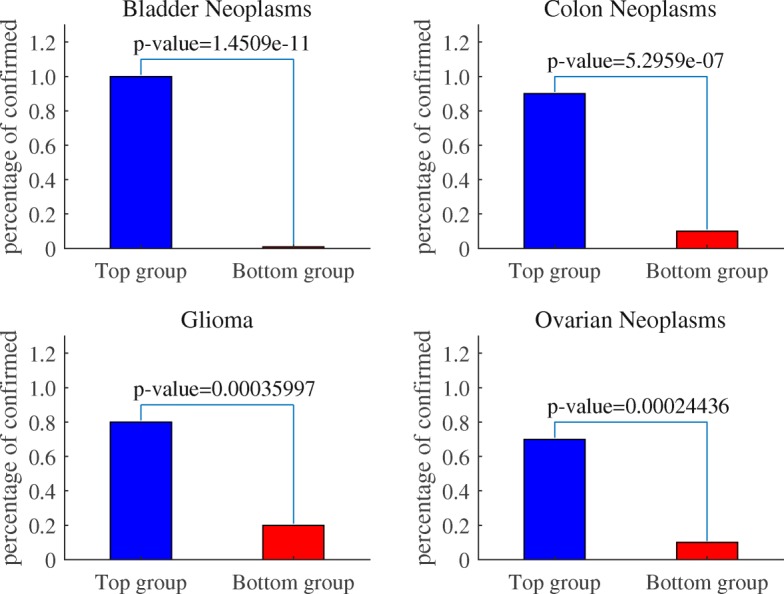
The percentage of confirmed candidate miRNAs in the Top group and Bottom group of the four diseases and the corresponding significance level of Fisher’s exact test

In addition, in order to further test the validity of the predicted results, we divided the candidate miRNAs for each disease into two groups according to the predicted scores, called Top group and Bottom group respectively [19], with 20 candidate miRNAs in each group, and then used fisher’s exact test to evaluate the statistical differences between the two groups. Figure 5 shows the proportion of confirmed candidate miRNAs in the Top group and Bottom group of four diseases and the significance level p by fisher’s exact test. For example, 18 of the candidate miRNAs in Colon Neoplasms’s Top group were confirmed (proportion of 0.9), and 2 of the Bottom group were confirmed (proportion of 0.1), with a *p* value of 5.2959 × 10^−7^. This suggests that the candidate miRNAs of Colon Neoplasms in the Top group are more likely to be confirmed than that in the Bottom group. Meanwhile, the *p* values were 1.4509 × 10^−11^ , 3.5997 × 10^−4^ , 2.4436 × 10^−4^ for Bladder Neoplasms, Glioma, Ovarian Neoplasms, respectively. The test results verified that the number of confirmed miRNAs in the Top group were significantly higher than that in the Bottom group, which further demonstrated the high efficiency of KNMBP algorithm in predicting new miRNA-disease interactions.

As shown in Additional file 5, the top 10 candidate miRNAs for these four diseases and their confirmation in HMDD v3.0 [31], miRCancer [37] and dbDEMC 2.0 [38]. Specifically, for Gladden Neoplasms and Colon Neoplasms, their top 10 candidate miRNAs were all confirmed in HMDD v3.0; For Glioma, 8 were confirmed in HMDD v3.0 and one was confirmed in miRCancer; For Ovarian Neoplasm, 9 were confirmed in HMDD v3.0 and one was confirmed in dbDEMC 2.0. Finally, all the interactions in Dataset II extracted from the current latest database were used as the training set, and the candidate miRNAs of 579 diseases predicted by KNMBP algorithm were sorted according to scores, as shown in Additional file 6.

## Discussion

The KNMBP proposed in this paper not only has high performance in predicting unknown miRNA-disease interactions, but also can efficiently predict the new miRNA (disease), which not associated with any disease (miRNA). In order to fairly evaluate the performance of the model, we compare the performance of it and several state-of-the-art models to the common Dataset (Dataset I) and the Dataset (Dataset II) extracted by ourselves for 5-fold cross validation (*CV*). In Dataset I, the AUC value of KNPMBP could reach 0.93126 when we perform *CV* on interactions. In Dataset II, the AUC value of KNMBP could reach 0.93795, 0.86937 and 0.86363 when we perform *CV* on interactions, on miRNAs and on diseases, respectively. The predicted results of our method were all better than other methods. In order to evaluate the predictive performance of KNMBP for new miRNA-disease interactions, we extracted the data from the old version database and tested the predicted results with the new version. Statistical results of 11 diseases confirmed that most of the top candidate miRNAs could be confirmed in the new version dataset. We divided the candidate miRNAs of the four common tumors into the Top group and the Bottom group according to the predicted scores. The fisher’s exact test results further confirmed that the number of confirmed miRNAs in the Top group were significantly higher than that in the Bottom group. In addition, the results of parameter sensitivity analysis show that KNMBP algorithm has the advantage of parameter robustness when the parameters are taken in a wide range.

The reason why the KNMBP algorithm has higher performance is mainly due to the following aspects. First, we constructed more reasonable disease semantic similarity network and miRNA functional similarity network. Specifically, instead of using Directed Acyclic Graph (DAG) alone to describe the disease similarity, we comprehensively used the gene-disease interactions, disease-GO biological process interactions and the MeSH descriptor to calculate the disease similarity, and more fully mined the similarity information between diseases to obtain more dense and accurate disease similarity network. In addition, previous methods for constructing miRNA functional similarity network mostly rely on the known miRNA-disease interaction, therefore they cannot predict new miRNAs. In this paper, the miRNA functional similarity is calculated by integrating miRNA-target gene interaction network and gene weight network, avoiding dependence on known miRNA-disease interactions and ensuring the prediction of new miRNAs. Secondly, in order to overcome the sparseness of the miRNA-disease interaction network and fully exploit the miRNA (disease) feature information, we utilized the weighted K neighborhood profiles to make a weighted correction on the sparse interaction network, taking advantage of neighborhood information to reduce the interaction network sparsity. Meanwhile, we used KSNS to calculate the miRNA (disease) kernel neighborhood similarity. Different from Gaussian function similarity and linear neighborhood similarity [20], KSNS not only makes full use of non-neighborhood information, but also fully excavates the nonlinear structural similarity between samples, consider both the distance similarity and the structural similarity of samples. Thirdly, we used diffusion component analysis to integrate the heterogeneous omics data of disease similarity and miRNA similarity respectively. The fused miRNA (disease) similarity network can not only effectively utilize the feature information among the known interactions, but also reflect the new similarity information obtained from other omics data. Fourthly, the bidirectional propagation algorithm simultaneously spreads the known miRNA-disease interactions from the similarity network of both disease and miRNA respectively, making full use of the global network information of miRNA and disease.

Although KNMBP efficiently predicted the unknown miRNA-disease interactions, there are some limitations. First, we tried to build the disease semantic similarity networks and miRNA functional similarity networks by making use of other latest data resources, however, there may be noises and errors in these similarity networks. Secondly, our evaluation is based on the known miRNA-disease interaction which may be not complete. Although the known miRNA-disease interactions have been greatly improved over the previous years, the proportion of these interaction in the total miRNA disease pair is still very low, which leads to some errors in the evaluation of our prediction results.

## Conclusion

Studies on the potential miRNA-disease interactions can help people understand the pathogenesis of diseases and design reasonable treatment schemes. In this paper, we proposed a new computational model (KNMBP) to predict the potential miRNA-disease interactions. Compared with other state-of-the-art methods, KNMBP not only has higher prediction accuracy on unknown miRNA-disease interaction, but also can effectively find potential interaction of new disease (or miRNA) without any known related miRNA (or disease). Furthermore, the proposed model is not sensitive to parameter. These indicate that our algorithm can integrate multiple omics data of miRNAs and diseases, and have a wide application prospect in miRNA and disease research.

## Supplementary information

**Additional file 1.** Details of the two benchmark data sets in the paper.

**Additional file 2.** The optimal parameters and the optimal AUC values of different experimental settings were performed on two benchmark data sets.

**Additional file 3.** The influence of non-neighborhood control parameter μ_1_ and similarity regularization parameter μ_2_ on the predictive performance of the model.

**Additional file 4.** The prediction scores of 199 new diseases and candidate mirnas sorted by score were obtained using the data set extracted from the old version HMDB.

**Additional file 5.** The top 10 candidate miRNAs of the four diseases predicted by KNMBP based on the old version.

**Additional file 6.** The candidate miRNAs of 579 diseases were sequenced according to the predicted score using the data set extracted from the new version of HMDB.

## Data Availability

The code and datasets are available at https://github.com/Mayingjun20179/KNMBP. The software is coded in Matlab in Windows system.

## Abbreviations

clusDCA: Improved Diffusion Component Analysis
DAG: Directed Acyclic Graph
KNMBP: Kernel neighborhood similarity and multi-network bidirectional propagation
KSNS: Kernel-based neighborhood similarity model
PLS: Partial least squares
SVM: Support vector machine
WKNNP: Weighted k-neighborhood profile

## References

[1] Filipowicz W, Bhattacharyya SN, Sonenberg N (2008). Mechanisms of post-transcriptional regulation by microRNAs: are the answers in sight?. Nat Rev Genet.

[2] Bartel DP (2009). MicroRNAs: target recognition and regulatory functions. Cell.

[3] Shabalina S, Koonin E (2008). Origins and evolution of eukaryotic RNA interference. Trends Ecol Evol.

[4] Guay C, Roggli E, Nesca V, Jacovetti C, Regazzi R (2011). Diabetes mellitus, a microRNA-related disease?. Transl Res.

[5] Nunez-Iglesias J, Liu CC, Morgan TE, Finch CE, Zhou XJ (2010). Joint genome-wide profiling of miRNA and mRNA expression in Alzheimer's disease cortex reveals altered miRNA regulation. PLoS One.

[6] Catto JWF, Alcaraz A, Bjartell AS, De Vere WR, Evans CP, Fussel S, Hamdy FC, Kallioniemi O, Mengual L, Schlomm T (2011). MicroRNA in prostate, bladder, and kidney Cancer: a systematic review. Eur Urol.

[7] Poy MN, Hausser J, Trajkovski M, Braun M, Collins S, Rorsman P, Zavolan M (2009). Stoffel M: miR-375 maintains normal pancreatic alpha- and beta-cell mass. Proc Natl Acad Sci U S A.

[8] Asangani IA, Rasheed SAK, Nikolova DA, Leupold JH, Colburn NH, Post S, Allgayer H (2008). MicroRNA-21 (miR-21) post-transcriptionally downregulates tumor suppressor Pdcd4 and stimulates invasion, intravasation and metastasis in colorectal cancer. Oncogene.

[9] Minn YK, Lee DH, Hyung WJ, Kim JE, Choi J, Yang SH, Song H, Lim BJ, Kim SH (2014). MicroRNA-200 family members and ZEB2 are associated with brain metastasis in gastric adenocarcinoma. Int J Oncol.

[10] Li Y, Zhang Z, Mao Y, Jin M, Jing F, Ye Z, Chen K (2014). A genetic variant in MiR-146a modifies digestive system Cancer risk: a meta-analysis. Asian Pac J Cancer Prev.

[11] Wang D, Wang J, Lu M, Song F, Cui Q (2010). Inferring the human microRNA functional similarity and functional network based on microRNA-associated diseases. Bioinformatics.

[12] Xu J, Li CX, Lv JY, Li YS, Xiao Y, Shao TT, Huo X, Li X, Zou Y, Han QL (2011). Prioritizing candidate disease miRNAs by topological features in the miRNA target-Dysregulated network: case study of prostate Cancer. Mol Cancer Ther.

[13] Chen X, Liu M, Yan G (2012). RWRMDA: predicting novel human microRNA–disease associations. Mol BioSyst.

[14] Chen X, Yan G (2015). Semi-supervised learning for potential human microRNA-disease associations inference. Sci Rep-UK.

[15] Chen X, Yang J, Guan N, Li J (2018). GRMDA: graph regression for MiRNA-disease association prediction. Front Physiol.

[16] Chen X, Xie D, Wang L, Zhao Q, You Z, Liu H (2018). BNPMDA: bipartite network projection for MiRNA–disease association prediction. Bioinformatics.

[17] Chen X (2018). WLQJ: predicting miRNA-disease association based on inductive matrix completion. Bioinformatics.

[18] Luo J, Xiao Q, Liang C, Ding P (2017). Predicting MicroRNA-disease associations using Kronecker regularized least squares based on heterogeneous Omics data. IEEE Access.

[19] Xiao Q, Luo J, Liang C, Cai J, Ding P (2018). A graph regularized non-negative matrix factorization method for identifying microRNA-disease associations. Bioinformatics.

[20] Zhang W, Qu Q, Zhang Y, Wang W (2018). The linear neighborhood propagation method for predicting long non-coding RNA–protein interactions. Neurocomputing.

[21] Li Y, Qiu C, Tu J, Geng B, Yang J, Jiang T, Cui Q (2013). HMDD v2.0: a database for experimentally supported human microRNA and disease associations. Nucleic Acids Res.

[22] Xuan P, Han K, Guo M, Guo Y, Li J, Ding J, Liu Y, Dai Q, Li J, Teng Z (2013). Prediction of microRNAs associated with human diseases based on weighted kMost similar neighbors. PLoS One.

[23] Lu M, Zhang Q, Deng M, Miao J, Guo Y, Gao W, Cui Q (2008). An analysis of human MicroRNA and disease associations. PLoS One.

[24] Davis AP, Grondin CJ, Johnson RJ, Sciaky D, McMorran R, Wiegers J, Wiegers TC, Mattingly CJ (2019). The comparative Toxicogenomics database: update 2019. Nucleic Acids Res.

[25] Karagkouni D, Paraskevopoulou MD, Chatzopoulos S, Vlachos IS, Tastsoglou S, Kanellos I, Papadimitriou D, Kavakiotis I, Maniou S, Skoufos G (2018). DIANA-TarBase v8: a decade-long collection of experimentally supported miRNA–gene interactions. Nucleic Acids Res.

[26] Chou C, Shrestha S, Yang C, Chang N, Lin Y, Liao K, Huang W, Sun T, Tu S, Lee W (2018). miRTarBase update 2018: a resource for experimentally validated microRNA-target interactions. Nucleic Acids Res.

[27] Hsu SD, Chu CH, Tsou AP, Chen SJ, Chen HC, PWC H, Wong YH, Chen YH, Chen GH, Huang HD (2007). miRNAMap 2.0: genomic maps of microRNAs in metazoan genomes. Nucleic Acids Res.

[28] Xiao F, Zuo Z, Cai G, Kang S, Gao X, Li T (2009). miRecords: an integrated resource for microRNA-target interactions. Nucleic Acids Res.

[29] Kozomara A, Birgaoanu M, Griffiths-Jones S (2019). miRBase: from microRNA sequences to function. Nucleic Acids Res.

[30] Lee I, Blom UM, Wang PI, Shim JE, Marcotte EM (2011). Prioritizing candidate disease genes by network-based boosting of genome-wide association data. Genome Res.

[31] Huang Z, Shi J, Gao Y, Cui C, Zhang S, Li J, Zhou Y, Cui Q (2019). HMDD v3.0: a database for experimentally supported human microRNA–disease associations. Nucleic Acids Res.

[32] Hu Y, Zhao T, Zhang N, Zang T, Zhang J, Cheng L (2018). Identifying diseases-related metabolites using random walk. BMC Bioinformatics.

[33] Deng L, Ye D, Zhao J, Zhang J. Exploring Disease Similarity by Integrating Multiple Data Sources. In: In 2018 IEEE International Conference on Bioinformatics and Biomedicine (BIBM). Madrid: IEEE; 2018. p. 853-58.

[34] Wang S, Cho H, Zhai C, Berger B, Peng J (2015). Exploiting ontology graph for predicting sparsely annotated gene function. Bioinformatics.

[35] Ma Y, Yu L, He T, Hu X, Jiang X (2018). Prediction of long non-coding RNA-protein interaction through kernel soft-neighborhood similarity. In 2018 IEEE international conference on Bioinformatics and biomedicine (BIBM).

[36] Liu Y, Wu M, Miao C, Zhao P, Li X (2016). Neighborhood regularized logistic matrix factorization for drug-target interaction prediction. PLoS Comput Biol.

[37] Xie B, Ding Q, Han H, Wu D (2013). miRCancer: a microRNA-cancer association database constructed by text mining on literature. Bioinformatics.

[38] Yang Z, Wu L, Wang A, Tang W, Zhao Y, Zhao H, Teschendorff AE (2017). dbDEMC 2.0: updated database of differentially expressed miRNAs in human cancers. Nucleic Acids Res.

