# Supplementary material for: MiRNA-disease interaction prediction based on kernel neighborhood similarity and multi-network bidirectional propagation
Source: BMC Med Genomics. 2019 Dec 23;12(Suppl 10):185. doi: 10.1186/s12920-019-0622-4 (PMC6927119; doi:10.1186/s12920-019-0622-4)
Supplement: Supplementary file 1 — Additional file 1. Details of the two benchmark data sets in the paper. [file 12920_2019_622_MOESM1_ESM.docx]

Additional file 1: Details of the two benchmark data sets in the paper.

| Benchmark dataset | miRNA-disease interaction | Disease information | miRNA information |
| --- | --- | --- | --- |
| Dataset I | 5430 interactions between 383 diseases and 495 miRNAs | two semantic similarity matrices of diseases | A miRNA functional similarity network |
| Dataset II | 10561 interactions between 574 miRNAs and 579 diseases | 1. 25,114,553 interactions between 46,045 genes and 7,163 diseases. 2. 1,727,119 interactions between 13126 GOs and 7116 diseases. 3. The name of the disease, MeSH ID, and parent IDs of 12988 diseases | 1. 588,134 interactions between 2814 miRNAs and 18468 genes 2. 476,399 interactions among 16243 genes |
