# Supplementary material for: MiRNA-disease interaction prediction based on kernel neighborhood similarity and multi-network bidirectional propagation
Source: BMC Med Genomics. 2019 Dec 23;12(Suppl 10):185. doi: 10.1186/s12920-019-0622-4 (PMC6927119; doi:10.1186/s12920-019-0622-4)
Supplement: Supplementary file 2 — Additional file 2. The optimal parameters and the optimal AUC values of different experimental settings were performed on two benchmark data sets. [file 12920_2019_622_MOESM2_ESM.docx]

Additional file 2: The optimal parameters and the optimal AUC values of different experimental settings were performed on two benchmark data sets.

| Benchmark dataset | CV | PN | $\lambda$($\lambda_{m}=\lambda_{d}$) | $\mu_{1}$ | $\mu_{2}$ | AUC |
| --- | --- | --- | --- | --- | --- | --- |
| Dataset I | CVa | 0.1 | 0.25 | 1 | 1 | 0.9313 |
| Dataset II | CVa | 0.1 | 0.25 | 1 | 1 | 0.9380 |
|  | CVd | 0.7 | 4 | 16 | 1 | 0.8636 |
|  | CVm | 0.1 | 2 | 16 | 2 | 0.8694 |
