# Supplementary material for: MiRNA-disease interaction prediction based on kernel neighborhood similarity and multi-network bidirectional propagation
Source: BMC Med Genomics. 2019 Dec 23;12(Suppl 10):185. doi: 10.1186/s12920-019-0622-4 (PMC6927119; doi:10.1186/s12920-019-0622-4)
Supplement: Supplementary file 5 — Additional file 5. The top 10 candidate miRNAs of the four diseases predicted by KNMBP based on the old version. [file 12920_2019_622_MOESM5_ESM.docx]

Additional file 5: The top 10 candidate miRNAs of the four diseases predicted by KNMBP based on the old version

| Disease | Number of  Confirmed | Top 10 predicted candidate miRNAs | | | | | |
| --- | --- | --- | --- | --- | --- | --- | --- |
|  |  | Rank | miRNA | Evidence | Rank | miRNA | Evidence |
| Bladder Neoplasms | 10 | 1  2  3  4  5 | hsa-mir-21  hsa-mir-17  hsa-mir-155  hsa-mir-20a  hsa-mir-18a | HMDD v3.0  HMDD v3.0  HMDD v3.0  HMDD v3.0  HMDD v3.0 | 5  6  7  9  10 | hsa-mir-145  hsa-mir-200b  hsa-mir-16-1  hsa-mir-19a  hsa-mir-146a | HMDD v3.0  HMDD v3.0  HMDD v3.0  HMDD v3.0  HMDD v3.0 |
| Colon Neoplasms | 10 | 1  2  3  4  5 | hsa-mir-21  hsa-mir-155  hsa-mir-20a  hsa-mir-18a  hsa-mir-16-1 | HMDD v3.0  HMDD v3.0  HMDD v3.0  HMDD v3.0  HMDD v3.0 | 5  6  7  8  9  10 | hsa-mir-19a  hsa-mir-146a  hsa-mir-29a  hsa-mir-34a  hsa-mir-143 | HMDD v3.0  HMDD v3.0  HMDD v3.0  HMDD v3.0  HMDD v3.0 |
| Glioma | 9 | 1  2  3  4  5 | hsa-mir-155  hsa-mir-19a  hsa-mir-146a  hsa-mir-15a  hsa-mir-29a | HMDD v3.0  HMDD v3.0  miRCancer  HMDD v3.0  HMDD v3.0 | 6  7  8  9  10 | hsa-mir-126  hsa-mir-148a  hsa-mir-210  hsa-let-7d  hsa-mir-223 | HMDD v3.0  HMDD v3.0  HMDD v3.0  unconfirmed  HMDD v3.0 |
| Ovarian Neoplasms | 10 | 1  2  3  4  5 | hsa-mir-143  hsa-mir-203  hsa-mir-15a  hsa-mir-210  hsa-mir-222 | HMDD v3.0  HMDD v3.0  dbDEMC 2.0  HMDD v3.0  HMDD v3.0 | 6  7  8  9  10 | hsa-mir-29c  hsa-mir-195  hsa-mir-150  hsa-mir-107  hsa-mir-193a | HMDD v3.0  HMDD v3.0  HMDD v3.0  HMDD v3.0  HMDD v3.0 |
